# Supplementary material for: Bone Marrow Macrophages Induce Inflammation by Efferocytosis of Apoptotic Prostate Cancer Cells via HIF-1α Stabilization
Source: Cells. 2022 Nov 22;11(23):3712. doi: 10.3390/cells11233712 (PMC9737180; doi:10.3390/cells11233712)
Supplement: Supplementary file 1 [file cells-11-03712-s001.zip › cells-1896328-supplementary.pdf]

Supplementary Table S1. Key Resources.

Species/ reagent type	Designation	Source of reference	Identifiers	Additional information
Mouse line	C57BL/6J	The Jackson Laboratory	C57BL/6J (Stock# 000664)	
Mouse line	FVB/NJ	The Jackson Laboratory	FVB/NJ (Stock# 001800)	
Mouse line	*Hif1a*^{mut}	The Jackson Laboratory	B6.129-*Hif1a*^{tm3Rsjo}/J (Stock# 007561)	Contains loxP sites flanking exon 2 of *Hif1a* (HIF-1α). Exposure to Cre recombinase removes the floxed sequence - creating a null allele.
Mouse line	*Epas1*^{mut}	The Jackson Laboratory	STOCK *Epas1*^{tm1Mcs}/J (Stock# 008407)	Contains loxP sites flanking exon 2 of *Epas1* (HIF-2α). Exposure to Cre recombinase results in exon 2 deletion.
Mouse line	LysMCre	The Jackson Laboratory	B6.129P2-*Lyz2*$^{tm1(cre)Ifo}$/J (Stock# 004781)	
Cell line (*Mus musculus*)	Prostate cancer, fibroblast-like (C57BL/6J)	ATCC	RM1 (ATCC, Cat.# CRL-3310)	Ras+Myc-induced prostate cancer that developed from a urogenital sinus mouse prostate reconstitution.
Cell line (*Mus musculus*)	Prostate cancer, epithelial-like (FVB/NJ)	ATCC	Myc-CaP (ATCC, Cat.# CRL-3255)	Derived from a genetically engineered mouse prostate cancer removed from an animal that was never exposed to hormone ablation.
Cell line (*Mus musculus*)	Primary Prostate Epithelial Cells (C57BL/6)	Cell Biologics	mPEC (Cell Biologics, Cat.#C57-6038)	Developed from prostate tissue of pathogen-free laboratory mice.
Probe	*18S* (*Mus musculus*)	ThermoFisher Scientific	TaqMan , assay ID Mm03928990_g1	
Probe	*Hif1a* (*Mus musculus*)	ThermoFisher Scientific	TaqMan , assay ID Mm00468869_m1	
Probe	*Epas1* (*Mus musculus*)	ThermoFisher Scientific	TaqMan , assay ID Mm01236108_m1	
Probe	*Adam8* (*Mus musculus*)	ThermoFisher Scientific	TaqMan , assay ID Mm01163449_g1	
Probe	*Hyou1* (*Mus musculus*)	ThermoFisher Scientific	TaqMan , assay ID Mm00491279_m1	
Probe	*Pdk1* (*Mus musculus*)	ThermoFisher Scientific	TaqMan , assay ID Mm00554300_m1	
Probe	*Stub1* (*Mus musculus*)	ThermoFisher Scientific	TaqMan , assay ID Mm00490634_m1	
Probe	*Cpbe1* (*Mus musculus*)	ThermoFisher Scientific	TaqMan , assay ID Mm01314928_m1	
Probe	*Pgk1* (*Mus musculus*)	ThermoFisher Scientific	TaqMan , assay ID Mm00435617_m1	
Probe	*Myc* (*Mus musculus*)	ThermoFisher Scientific	TaqMan , assay ID Mm00487804_m1	
Probe	*Ldha* (*Mus musculus*)	ThermoFisher Scientific	TaqMan , assay ID Mm01612132_g1	
Probe	*Mif* (*Mus musculus*)	ThermoFisher Scientific	TaqMan , assay ID Mm01611157_gH	
Probe	*Cd74* (*Mus musculus*)	ThermoFisher Scientific	TaqMan , assay ID Mm00658576_m1	
Probe	*Cxcl1* (*Mus musculus*)	ThermoFisher Scientific	TaqMan , assay ID Mm04207460_m1	
Probe	*Pf4* (*Mus musculus)*	ThermoFisher Scientific	TaqMan , assay ID Mm00451315_g1	
Probe	*Cxcl5* (*Mus musculus*)	ThermoFisher Scientific	TaqMan , assay ID Mm00436451_g1	
Probe	*Il6* (*Mus musculus*)	ThermoFisher Scientific	TaqMan , assay ID Mm00446190_m1	
Probe	*Ccl2* (*Mus musculus*)	ThermoFisher Scientific	TaqMan , assay ID Mm00441242_m1	
Probe	*Ccl5* (*Mus musculus*)	ThermoFisher Scientific	TaqMan , assay ID Mm01302427_m1	
Probe	*Il1b (Mus musculus)*	ThermoFisher Scientific	TaqMan , assay ID Mm00434228_m1	
Probe	*Cd80 (Mus musculus)*	ThermoFisher Scientific	TaqMan , assay ID Mm00711660_m1	
Probe	*Glut1 (Mus musculus)*	ThermoFisher Scientific	TaqMan , assay ID Mm00441480_m1	
Probe	*Tnf (Mus musculus)*	ThermoFisher Scientific	TaqMan , assay ID Mm00443258_m1	
Probe	*Il10 (Mus musculus)*	ThermoFisher Scientific	TaqMan , assay ID Mm01288386_m1	
Probe	*Arg1 (Mus musculus)*	ThermoFisher Scientific	TaqMan , assay ID Mm00475988_m1	
Probe	*Cd36 (Mus musculus)*	ThermoFisher Scientific	TaqMan , assay ID Mm00432403_m1	
Probe	*Cd206 (Mus musculus)*	ThermoFisher Scientific	TaqMan , assay ID Mm01329359_m1	
Primers	B6.129-*Hif1a*^{tm3Rsjo}/J (genotyping)	ThermoFisher Scientific	Fw: 5'-TGCATGTGTATGGGTGTTTTG-3' Rv: 5'-GAAAACTGTCTGTAACTTCATTTCC-3'	

Primers	STOCK *Epas1*^{tm1Mcs}/J (genotyping)	ThermoFisher Scientific	Fw: 5'-GAGAGCAGCTTCTCCTGGAA-3'<br>Rv: 5'-TGTAGGCAAGGAAACCAAGG-3'	
Primers	B6.129P2-*Lyz2*$^{tm1(cre)Ifo}$/J (genotyping)	ThermoFisher Scientific	Mutant: 5'-CCCAGAAATGCCAGATTACG-3'<br>Common: 5'-CTTGGGCTGCCAGAATTTCTC-3’<br>Wildtype: 5'-TTACAGTCGGCCAGGCTGAC-3'	
Antibody	Anti HIF-1α (D1S7W) XP, Rabbitmonoclonal	Cell Signaling Technology	Cat.# 36169	WB (1:2000); IP (1:50)
Antibody	Anti MIF, Rabbit polyclonal	Cell Signaling Technology	Cat.# 88186	WB (1:3000)
Antibody	Anti-phospho Stat3 (Tyr705) (D3A7) XP®, Rabbit monoclonal	Cell Signaling Technology	Cat.# 9145	WB (1:3000)
Antibody	Anti-phospho Stat3 (Tyr705) (3E2), Mouse monoclonal	Cell Signaling Technology	Cat.# 9138	WB (1:2000)
Antibody	Anti p44/42 MAPK (Erk1/2) (137F5), Rabbit monoclonal	Cell Signaling Technology	Cat.# 4695	WB (1:3000)
Antibody	Anti-phospho p44/42 MAPK (Erk1/2) (Thr202/Tyr204) (D13.14.4E) XP®, Rabbit monoclonal	Cell Signaling Technology	Cat.# 4370	WB (1:3000)
Antibody	Anti NFκB p65 (D14E12) XP®, Rabbit monoclonal	Cell Signaling Technology	Cat.# 8242	WB (1:3000)
Antibody	Anti-phospho NF-κB p65 (Ser536) (93H1), Rabbit monoclonal	Cell Signaling Technology	Cat.# 3033	WB (1:3000)
Antibody	APC Anti-F4/80 antibody [CI:A3-1] (Allophycocyanin)	Abcam	Cat.# ab105080	FC (1:100)
Antibody	APC Rat IgG2b, kappa monoclonal [KLH/G2b-1-2] - Isotype control (Allophycocyanin)	Abcam	Cat.# ab154434	FC (1:100)
Antibody	FITC Anti-F4/80 [Cl:A3-1]	BioRad	Cat.# MCA497FB	FC (1:100)
Antibody	FITC Rat IgG2b, Isotype control	BioRad	Cat.# MCA6006	FC (1:100)
